# Psychopathological profiles in offenders with antisocial personality disorder: a comparison of violent and sexual crimes

**DOI:** 10.1007/s11845-026-04285-3

**Published:** 2026-02-18

**Authors:** Tuba Özcanlı, Muhammed Emin Boylu, Yasin Kavla, Ömer Asan, Emre Çırakoğlu

**Affiliations:** 1 Council of Forensic Medicine, Ministry of Justice, İstanbul, Türkiye; 2 Department of Psychiatry, Cerrahpaşa Faculty of Medicine, İstanbul University – Cerrahpaşa, İstanbul, Türkiye

**Keywords:** Cognitive distortions, Forensic psychiatry, Psychopathy, Risk assessment, Substance use

## Abstract

**Introduction:**

This study examines psychopathy and aggression profiles in offenders with Antisocial Personality Disorder (ASPD) using standardized forensic assessment tools. It hypothesizes that higher psychopathy scores (PCL-R) will be significantly associated with greater aggression, providing insights for forensic risk evaluation and management.

**Method:**

The study included incarcerated offenders evaluated at the Council of Forensic Medicine between 2014 and 2024 who were diagnosed with ASPD according to DSM-5 criteria. Participants voluntarily completed standardized assessments including the Psychopathy Checklist–Revised (PCL-R), Buss–Perry Aggression Questionnaire (BPAQ), Beck Depression and Anxiety Inventories, and the Interpersonal Cognitive Distortions Scale. Sociodemographic, clinical, and forensic data were collected through a semi-structured interview form and official court records. Group comparisons and correlation analyses were performed, with statistical significance set at *p* < 0.05.

**Results:**

Among offenders with ASPD, violent offenders were more frequently unemployed (58.8%) and divorced (45.1%) compared to sexual offenders (31.6% and 18.4%, respectively; *p* = 0.009 and *p* = 0.022). Substance use (84.3% vs. 44.7%; *p* < 0.001), non-suicidal self-injury (86.3% vs. 28.9%; *p* < 0.001), and physical abuse (88.2% vs. 44.7%; *p* < 0.001) were markedly higher in the violent group, while sexual offenders more often reported sexual abuse (52.6%; *p* < 0.001). Psychopathy scores were significantly higher in violent offenders (Mean = 28.0 ± 5.8) than sexual offenders (Mean = 11.9 ± 3.7; *p* < 0.001), whereas depression scores were greater in sexual offenders (Mean = 35.2 ± 8.1 vs. 13.6 ± 5.3; *p* < 0.001). Logistic regression identified psychopathy as the strongest predictor of violent offending (β = 0.60, *p* < 0.001, OR = 1.82), explaining 83% of variance (Nagelkerke R² = 0.83).

**Conclusion:**

Violent and sexual offenders with ASPD exhibited distinct profiles, with psychopathy strongly associated with violent offending and internalizing symptoms and sexual trauma linked to sexual crimes. These findings emphasize the need for individualized, mechanism-based forensic interventions and risk assessments focusing on psychopathy and cognitive factors.

## Introduction

Antisocial Personality Disorder (ASPD) is characterized by a pervasive pattern of disregard for and violation of the rights of others, deceitfulness, impulsivity, and a lack of remorse or empathy. Although individuals with ASPD constitute only a small fraction of the general population, they represent a disproportionately large segment of incarcerated offenders and are responsible for a considerable share of violent crimes and recidivism [1, 2]. ASPD has been linked to a constellation of biological, psychological, and social factors, including early childhood adversity, neurobiological abnormalities, and environmental stressors [3, 4]. Understanding the behavioral and cognitive correlates of ASPD is therefore crucial for forensic psychiatry, where accurate assessment of responsibility, risk, and treatment needs remains a central task.

The construct of psychopathy, while conceptually overlapping with ASPD, is regarded as a distinct clinical entity characterized by affective and interpersonal deficits such as callousness, superficial charm, egocentricity, and manipulativeness [1, 5, 6]. Psychopathy is commonly assessed using the Psychopathy Checklist-Revised (PCL-R), which provides a dimensional measure of these traits. Research consistently demonstrates that psychopathy is strongly associated with instrumental aggression, violent offending, and poor response to conventional treatment [7, 8]. However, the degree to which psychopathic traits contribute to aggression in offenders with a formal diagnosis of ASPD remains insufficiently explored, especially in forensic populations undergoing psychiatric evaluation.

Aggression in ASPD and psychopathy manifests in both reactive (impulsive, emotion-driven) and instrumental (planned, goal-directed) forms [9, 10]. Neurocognitive models propose that deficits in amygdala-mediated emotional learning and prefrontal inhibitory control may underpin these behaviors [11]. Functional neuroimaging and electrophysiological studies have shown altered activation in the prefrontal cortex, amygdala, and orbitofrontal regions among individuals with psychopathic and antisocial traits [12, 13]. Such findings suggest that aggression in ASPD cannot be fully understood without accounting for underlying psychopathic characteristics that modulate emotional regulation and moral reasoning.

Despite substantial theoretical work, empirical studies examining both psychopathy and aggression levels specifically within incarcerated individuals formally diagnosed with ASPD remain scarce. Most available research either focuses on psychopathy irrespective of DSM-based diagnoses or investigates aggression in mixed offender samples [14]. Moreover, the heterogeneity of ASPD presentations — often influenced by comorbid substance use or mood disorders — further complicates the understanding of aggression mechanisms in this population. There is therefore a pressing need for comprehensive, data-driven research that integrates standardized forensic psychiatric assessments with validated psychometric measures to clarify the psychopathy–aggression relationship in ASPD.

The present study aims to investigate the levels of psychopathy and aggression among incarcerated offenders diagnosed with ASPD and to examine the relationship between these constructs using standardized forensic assessment data. We hypothesize that higher psychopathy scores, as measured by the PCL-R, will be significantly associated with greater levels of aggression on psychometric scales. This relationship, if confirmed, could have implications for forensic risk assessment, treatment planning, and violence prevention strategies within correctional and psychiatric settings.

## Method

### Participants

The study population consisted of incarcerated offenders evaluated at the Observation and Assessment Department of the Council of Forensic Medicine (Adli Tıp Kurumu Gözlem İhtisas Dairesi) between March 1, 2014, and March 1, 2024. Eligible participants were those diagnosed with ASPD according to DSM-5 criteria and who voluntarily consented to undergo psychometric testing as part of their forensic psychiatric examination. Inclusion criteria included: [1] a confirmed ASPD diagnosis by board-certified forensic psychiatrists; [2] completion of all psychometric instruments listed below; and [3] cognitive capacity to provide informed consent. Exclusion criteria comprised acute psychosis, major neurocognitive disorder, or intellectual disability that could compromise test validity.

Sociodemographic characteristics recorded for each participant included age, educational attainment, marital status, employment, economic status, and residential background (urban, semi-urban, rural). Clinical and familial data encompassed history of psychiatric treatment, family psychiatric history, substance and alcohol use, self-mutilation, suicidal behavior (presence, method, number of attempts), family cohesion, parental divorce, trauma exposure (physical, emotional, sexual abuse), and migration history. Forensic data included type of offense (e.g., theft, robbery, assault, attempted murder, homicide, sexual offenses, threat/insult, firearm possession, escape, abduction, and other crimes), severity of crime (violent vs. non-violent), age at first offense, length of imprisonment, and total number of convictions. Because psychometric tests were not uniformly applied in every case, a 10-year observation window was selected to achieve a representative sample size.

### Procedure

Each case was identified and reviewed through the institutional digital archive and paper-based forensic reports. Diagnoses were established by multidisciplinary teams using DSM-5 criteria, combining clinical observation, collateral interviews, and criminal record review during the standard 3–4-week observation period. All participants completed a standardized psychometric battery under the supervision of certified forensic psychologists, consisting of: Psychopathy Checklist–Revised (PCL-R; Hare, 2003) — assessing interpersonal, affective, lifestyle, and antisocial facets of psychopathy. Aggression Questionnaire (Buss & Perry, 1992) — measuring total and subscale aggression (physical, verbal, anger, hostility). Beck Depression Inventory (BDI) and Beck Anxiety Inventory (BAI) — evaluating mood and anxiety symptoms (Beck et al., 1996). Cognitive Distortions in Interpersonal Relationships Scale — assessing maladaptive cognitions in social interactions (e.g., mistrust, overgeneralization, self-blame). Semi-Structured Interview Form — developed by the research team to capture clinical and sociodemographic data. Additionally, each participant’s official court file was examined to obtain judicial and contextual variables, including criminal background, nature and motivation of offenses, substance use patterns, psychiatric and medical comorbidities, and forensic psychiatric history. All participants completed the Minnesota Multiphasic Personality Inventory (MMPI) as part of their forensic psychiatric evaluation, and the ten clinical scales (Hypochondriasis, Depression, Hysteria, Psychopathic Deviate, Masculinity–Femininity, Paranoia, Psychasthenia, Schizophrenia, Hypomania, and Social Introversion) were included in the analyses.

All personally identifiable information was removed before analysis. The study was approved by the Scientific Research Ethics Committee of the Council of Forensic Medicine (03.12.2024 / 21589509/2024/1418), and conducted in accordance with the Declaration of Helsinki.

### Data collection tools

#### Psychopathy checklist–revised (PCL-R; Hare, 2003)

The PCL-R is a 20-item rating instrument designed for use in research, clinical, and forensic settings. Each item is scored 0 (does not apply), 1 (applies somewhat), or 2 (definitely applies) on the basis of a semi-structured interview, file review, and collateral information. The scale assesses psychopathic personality traits across four facets: interpersonal, affective, lifestyle, and antisocial behaviour. Higher total scores reflect greater expression of psychopathic traits. Numerous studies attest to acceptable reliability (e.g., ICC ~ 0.86 for male criminal offenders) and predictive validity for antisocial and violent behaviour.

#### Aggression questionnaire (Buss & Perry, 1992)

The Buss-Perry Aggression Questionnaire (BPAQ) is a self-report instrument consisting of 29 items rated on a Likert scale (typically 1 = “extremely uncharacteristic of me” to 5 = “extremely characteristic of me”). It measures four dimensions of aggression: Physical Aggression, Verbal Aggression, Anger, and Hostility. The internal structure, reliability, and validity of the BPAQ have been established across various populations, though its generalizability to forensic populations has been examined with caution. Higher scores indicate higher levels of aggressive disposition and behaviours.

#### Beck depression inventory (BDI) & beck anxiety inventory (BAI)

The BDI and BAI are widely used self-report measures to assess the severity of depressive and anxiety symptoms, respectively. The BDI (21 items) evaluates symptoms of depression such as sadness, hopelessness, and self-criticism. The BAI (21 items) assesses anxiety symptoms such as nervousness, fear, and somatic symptoms. Both use a 0–3 response format per item. Validity and reliability have been well documented in clinical and non-clinical settings.

#### Interpersonal cognitive distortions scale (ICDS; Hamamcı & Büyüköztürk, 2004)

The ICDS is a measure developed to assess cognitive distortions in interpersonal relationships. The scale was constructed on a sample of university students (*N* ≈ 425) and a factor analysis identified three subscales: Interpersonal Rejection, Unrealistic Relationship Expectations, and Interpersonal Misperception. The test-retest reliability of the total scale was reported as *r* ≈ 0.74, and internal consistency coefficients ranged acceptable for two of the subscales (e.g., 0.70, 0.76) though one subscale (Misperception) had weaker reliability. The ICDS has been used to explore the relationship between interpersonal cognitive distortions and other psychological constructs.

#### Semi-structured interview form (Researchers’ Instrument)

A semi-structured interview form was developed by the research team to systematically collect sociodemographic, clinical, forensic and trauma-related information from each participant. Key variables included: age, educational attainment, marital status, employment, economic status, residential status, prior psychiatric treatment, family psychiatric history, substance/alcohol use, self-mutilation history, suicidal behaviour (attempt history and method), family structure (e.g., parental divorce), trauma exposure (physical/emotional/sexual abuse), migration history, age at first offence, number and type of offences, crime severity, length of imprisonment, and medical comorbidities. These interview data were supplemented by file review of court dossiers and institutional records.

### Statistical analyses

Statistical analyses were conducted using IBM SPSS Statistics Version 26.0 (IBM Corp., Armonk, NY, USA). Descriptive statistics (mean ± standard deviation, frequency, percentage) summarized demographic, clinical, and forensic characteristics. The Kolmogorov–Smirnov test was used to assess normality of continuous variables. Group comparisons (e.g., high vs. low psychopathy scores) employed independent-samples t-tests or Mann–Whitney U tests, as appropriate. Relationships among psychopathy (PCL-R), aggression, depression, anxiety, cognitive distortions, and MMPI clinical scales were examined using Pearson’s or Spearman’s correlation coefficients. Predictors of aggression were explored using multiple linear regression analyses, entering psychopathy scores, substance use, number of offenses, and affective symptoms as independent variables. For secondary analyses, logistic regression was applied to identify variables associated with violent crime (vs. non-violent). Statistical significance was defined as *p* < 0.05 (two-tailed).

## Results

Among the examined variables, working status and marital status showed statistically significant differences between violent and sexual offenders. A greater proportion of violent offenders were unemployed (58.8%), whereas sexual offenders more frequently reported regular or irregular employment (28.9% and 39.5%, respectively; *p* = 0.009, χ² = 9.408). Regarding marital status, divorced individuals were more common among violent offenders (45.1%) compared to sexual offenders (18.4%), while single individuals were more prevalent in the sexual crime group (65.8% vs. 39.2%; *p* = 0.022, χ² = 7.639). No significant differences were observed in education level, economic status, family relationships, parental marital status, living place, or migration history (*p* > 0.05). Details are given in Table 1.

**Table 1:** Sociodemographic features of violent and sexual crime offenders

		Crime type					
		Violent crime		Sexual crime		p*	X^2^
*n*	*n*%	*n*	*n*%	*n*	*n*%		
Education Status	> 8 Years	25	49,0%	15	39,5%	0.371	0.802
	< 8 Years	26	51,0%	23	60,5%		
Marital Status	Married	8	15,7%	6	15,8%	*0.022*	7.639
	Single	20	39,2%	25	65,8%		
	Divorced	23	45,1%	7	18,4%		
Working Status	Not working	30	58,8%	12	31,6%	*0.009*	9.408
	Regular working	4	7,8%	11	28,9%		
	Irregular working	17	33,3%	15	39,5%		
Economic Status	Low	28	54,9%	16	42,1%	0.286	1.427
	Moderate and High	23	45,1%	22	57,9%		
Family Relationship	No	33	64,7%	20	52,6%	0.281	1.318
	Yes	18	35,3%	18	47,4%		
Parents Marital Status	Married	18	35,3%	7	18,4%	0.098	3.069
	Divorced	33	64,7%	31	81,6%		
Living Place	City	42	82,4%	28	73,7%	0.587	1.067
	Town	6	11,8%	6	15,8%		
	Village	3	5,9%	4	10,5%		
İmmigration History	No	9	17,6%	10	26,3%	0.434	0.975
	Yes	42	82,4%	28	73,7%		
Age (Mean SD)							

*X2* Chi-Square *U Mann Whitney U test

Significant differences were found in substance use, non-suicidal self-injury, trauma type, and psychiatric treatment history between violent and sexual offenders. Substance use was markedly higher among violent offenders (84.3%) than sexual offenders (44.7%; *p* < 0.001, χ² = 15.527). Similarly, non-suicidal self-injury was much more common in the violent crime group (86.3% vs. 28.9%; *p* < 0.001, χ² = 30.313). Regarding trauma exposure, violent offenders predominantly reported physical abuse (88.2%), whereas sexual offenders more frequently reported sexual abuse (52.6%; *p* < 0.001, χ² = 22.220). In addition, a higher proportion of violent offenders had received previous psychiatric treatment (64.7% vs. 39.5%; *p* = 0.018, χ² = 5.580). No significant differences were observed between the groups in terms of psychiatric history, family psychiatric history, alcohol use, suicide attempts, suicide method, or reason for psychiatric referral (*p* > 0.05). Details are given in Table 2.

**Table 2:** Clinical and criminological features of violent and sexual crime offenders

		Crime type					
		Violent crime		Sexual crime		p*	X^2^
*n*	*n*%	*n*	*n*%	*n*	*n*%		
Psychiatric History	No	8	15,7%	11	28,9%	0.131	2.281
	Yes	43	84,3%	27	71,1%		
Family Psychiatric History	No	27	52,9%	21	55,3%	0.828	0.047
	Yes	24	47,1%	17	44,7%		
Alcohol Use	No	10	19,6%	3	7,9%	0.122	2.395
	Yes	41	80,4%	35	92,1%		
Substance Use	No	8	15,7%	21	55,3%	0.001	15.527
	Yes	43	84,3%	17	44,7%		
Non-Suicidal Self Injury	No	7	13,7%	27	71,1%	0.001	30.313
	Yes	44	86,3%	11	28,9%		
Trauma Type	No	2	3,9%	1	2,6%	0.001	22.220
	Physical Abuse	45	88,2%	17	44,7%		
	Sexual Abuse	4	7,8%	20	52,6%		
Suicide Attempt	No	18	35,3%	17	44,7%	0.367	0.814
	Yes	33	64,7%	21	55,3%		
Suicide Type	Yok	17	33,3%	17	44,7%	0.070	7.070
	Drug Overdose	23	45,1%	15	39,5%		
	Jump From High	1	2,0%	4	10,5%		
	Firearm, Hanging, Cutting	10	19,6%	2	5,3%		
Treatment	No	18	35,3%	23	60,5%	0.018	5.580
	Yes	33	64,7%	15	39,5%		
Reason for Psychiatric Referral	Substance Abuse	13	25,5%	7	18,4%	0.716	0.668
	Adjustment Disorder	15	29,4%	13	34,2%		
	Forensic Reasons	23	45,1%	18	47,4%		
Criminal Record	No	11	21,6%	3	7,9%	0.080	3.71
	Yes	40	78,4%	35	92,1%		
First Crime Age (Mean SD)		16.19 ± 2.72		15.47 ± 2.16		0.181	1.124

*X2* Chi-Square *U Mann Whitney U test

Significant differences were observed between violent and sexual offenders across several psychometric measures. Psychopathy scores (PCL-R) were markedly higher among violent offenders (Mean = 28.01 ± 5.83) compared to sexual offenders (Mean = 11.94 ± 3.70; *p* < 0.001, t = 14.878), indicating stronger psychopathic traits in the violent crime group. Conversely, depressive symptoms (BDI) were substantially higher in sexual offenders (Mean = 35.15 ± 8.06) than in violent offenders (Mean = 13.64 ± 5.28; *p* < 0.001, t = − 15.183). Sexual offenders also showed higher anxiety scores (*p* = 0.01, t = − 2.617), MMPI Hysteria (*p* = 0.002, t = − 3.214), Masculinity–Femininity (MF) (*p* < 0.001, t = − 3.322), Paranoia (*p* = 0.002, t = − 3.247), Psychasthenia (*p* = 0.009, t = − 1.714), Hypomania (*p* = 0.03, t = − 2.209), and Social Introversion (*p* = 0.004, t = − 2.977) scores. In contrast, violent offenders exhibited significantly higher scores on the Cognitive Distortions Scale (*p* < 0.001, t = 4.977), Hypochondriasis (*p* = 0.02, t = 2.375), Psychopathic Deviate (*p* = 0.014, t = 2.518), and Schizophrenia (*p* < 0.001, t = 7.347) scales, suggesting greater cognitive distortion and psychotic-like thinking tendencies. No significant difference was found for the MMPI Depression subscale (*p* = 0.268). These results indicate distinct psychological and psychopathological profiles between violent and sexual offenders, as summarized in Table 3.

**Table 3:** Psychometric scale scores in violent and sexual offenders

Scale	Violent crime (*n* = 51)		Sexual crime (*n* = 38)		*p*	t
	Mean	SD	Mean	SD		
PCL-R (Hare Score)	28.01	5.83	11.94	3.70	0.001	14,878
Beck Depression Inventory	13.64	5.28	35.15	8.06	0.001	-15,183
Beck Anxiety Inventory	13.60	6.87	17.00	4.70	0.01	-2,617
Cognitive Distortions Scale	34.64	7.13	27.54	5.95	0.001	4,977
MMPI – Hypochondriasis	37.31	10.27	32.05	10.41	0.02	2,375
MMPI – Depression	19.19	4.61	17.76	7.46	0.268	1,115
MMPI – Hysteria	24.78	8.33	31.00	9.88	0.002	-3,214
MMPI – Psychopathic Deviate	30.05	8.90	25.50	7.79	0.014	2,518
MMPI – Masculinity–Femininity (MF)	35.56	15.85	45.42	10.51	0.001	-3,322
MMPI – Paranoia	23.31	7.76	29.39	9.90	0.002	-3,247
MMPI – Psychasthenia	21.86	8.56	24.73	6.69	0.009	-1,714
MMPI – Schizophrenia	37.66	12.35	22.39	3.87	0.001	7,347
MMPI – Hypomania	16.23	3.58	17.78	2.82	0.03	-2,209
MMPI – Social Introversion	33.09	7.41	37.86	7.55	0.004	-2,977

*t*: Simple t Test

A binary logistic regression analysis was conducted to identify predictors of violent versus sexual offending among individuals diagnosed with ASPD. The dependent variable was crime type (1 = violent, 0 = sexual). Independent variables included psychopathy (PCL-R score), cognitive distortions, substance use, and anxiety level (Beck Anxiety Inventory). The overall model was statistically significant, χ²(4, *N* = 89) = 100.93, *p* < 0.001, with a Nagelkerke R² of 0.83, indicating that approximately 83% of the variance in offense type was explained by the predictors. Among the variables, psychopathy score (PCL-R) emerged as a strong and statistically significant predictor of violent offending (β = 0.60, *p* < 0.001). This indicates that each one-point increase in psychopathy score was associated with approximately a 1.82-fold increase in the odds of committing a violent crime (OR = e^0.60 = 1.82, 95% CI [1.31, 2.54]). Cognitive distortions were marginally associated with violent offending (β = 0.31, *p* = 0.069), suggesting a trend-level effect. Substance use (β = 1.97, *p* = 0.215) and anxiety levels (β = 0.15, *p* = 0.146) did not significantly predict offense type. These findings indicate that higher psychopathy scores substantially increase the likelihood of violent offending, whereas cognitive distortions may have a secondary or mediating role. Anxiety and substance use were not independent predictors once psychopathy was controlled for. Details are presented in Table 4.

**Table 4:** Logistic regression predicting violent crime among offenders with ASPD

Variable	β	Std. error	z	*p*	95% GA
Constant	–24.15	7.87	–3.07	0.002	[–39.57, − 8.72]
Hare_Score (PCL-R)	0.60	0.17	3.57	< 0.001	[0.27, 0.93]
Cognitive Dist.	0.31	0.17	1.82	0.069	[–0.02, 0.65]
Substance Use	1.97	1.59	1.24	0.215	[–1.14, 5.07]
Beck Anxiety	0.15	0.10	1.45	0.146	[–0.05, 0.35]

## Discussion

This study compared sociodemographic, clinical, and psychometric characteristics between violent and sexual offenders diagnosed with ASPD who underwent forensic psychiatric evaluation. The findings revealed distinct patterns across psychopathy, affective symptoms, cognitive distortions, and criminal background, providing valuable insights into heterogeneity within ASPD and its forensic relevance.

No significant group differences were found in education, economic level, family relationships, or residential background, suggesting that basic demographic variables alone are insufficient to distinguish violent from sexual offenders. This finding aligns with prior research indicating that while low socioeconomic status, limited education, and unstable employment are general risk factors for criminality, they do not meaningfully differentiate offense types among individuals with ASPD [15, 16]. The majority of participants, regardless of offense category, exhibited early social disadvantage and fragmented family structures, reflecting the cumulative impact of adverse developmental conditions on antisocial trajectories.

In contrast, working status and marital status differed significantly between groups: violent offenders were more often unemployed and divorced, whereas sexual offenders were more likely to report irregular work and to be single. Prior studies have suggested that marital disruption and occupational instability are linked to impulsive aggression and interpersonal dysfunction in violent offenders [17], while social isolation and unmet intimacy needs may characterize sexual offending [18].

Criminal history variables showed a clear divergence consistent with theoretical models of ASPD. Violent offenders exhibited more extensive prior convictions, earlier onset of offending, and a higher rate of physical aggression. This aligns with evidence that early criminal onset and chronic offending patterns are characteristic of high-psychopathy individuals with elevated impulsivity and callous-unemotional traits [19]. Conversely, sexual offenders often demonstrate fewer total offenses but higher specialization in sexual crime, consistent with more internally driven pathology rather than generalized antisociality [20].

Clinically, violent offenders showed higher rates of substance use, non-suicidal self-injury, and physical trauma history, whereas sexual offenders reported more frequent sexual abuse histories and higher depression and anxiety scores. The strong association between substance use and violent offending in ASPD has been well-documented, as intoxication and withdrawal states can amplify impulsivity and reduce inhibition [21]. Similarly, the prevalence of self-harm among violent offenders may reflect dysregulated affect and impulsive coping mechanisms, features commonly observed in severe ASPD phenotypes [22].

The link between sexual abuse history and sexual offending observed in this study corroborates prior findings that early traumatic sexual experiences can distort sexual scripts and attachment representations, increasing risk for later deviant sexual behavior [23]. Moreover, the higher prevalence of depressive and anxious symptoms among sexual offenders echoes research indicating that sexual offending is often accompanied by internalizing psychopathology, guilt, and shame [24]. These affective states may represent both risk factors and targets for therapeutic intervention.

One of the most robust findings of this study was the marked difference in psychopathy levels between the two offender groups. Violent offenders had significantly higher PCL-R scores, and logistic regression confirmed psychopathy as the strongest independent predictor of violent crime (β = 0.60, *p* < 0.001). This supports a large body of research linking psychopathy with instrumental aggression, emotional callousness, and recidivism [25–27]. The implication is that within ASPD, psychopathy represents a distinct, more malignant subtype characterized by predatory aggression and diminished empathy.

Cognitive distortions were also more pronounced among violent offenders, consistent with cognitive-behavioral models positing that maladaptive schemas (e.g., hostility, mistrust, and externalization of blame) mediate the relationship between personality pathology and violent behavior [28]. While this association was only marginally significant in regression analysis (*p* = 0.069), the pattern suggests that cognitive restructuring may be a valuable therapeutic target in violent ASPD offenders.

The MMPI results revealed distinct psychopathological profiles across offense types. Violent offenders scored higher on the Psychopathic Deviate and Schizophrenia subscales, indicating greater behavioral dysregulation, emotional blunting, and psychotic-like thought patterns. Sexual offenders, by contrast, scored higher on Hysteria, Paranoia, Masculinity–Femininity, and Social Introversion, reflecting heightened emotional sensitivity, social withdrawal, and possible identity-related conflicts. These findings correspond to prior reports showing that sexual offenders tend to exhibit more neurotic and introverted features, whereas violent offenders show higher externalizing pathology and impulsivity [29, 30]. The coexistence of psychopathy and psychotic-like features among violent offenders also supports neurobiological theories emphasizing dysfunction in amygdala-prefrontal circuits underlying deficient emotional regulation and moral reasoning [31, 32].

The logistic regression model explained a substantial portion of the variance in offense type (Nagelkerke R² = 0.83). Psychopathy emerged as the strongest predictor of violent offending, while cognitive distortions showed a trend-level association. Substance use and anxiety did not independently predict crime type once psychopathy was accounted for. This pattern supports the notion that psychopathy operates as a central organizing construct within ASPD, integrating affective, behavioral, and cognitive deficits that predispose to violence [22]. The high explanatory power of the model demonstrates that a small number of clinically measurable constructs—psychopathy and maladaptive cognition—can meaningfully discriminate between violent and sexual offending trajectories among individuals with ASPD. This has direct implications for risk assessment and individualized treatment planning in forensic psychiatry.

The Nagelkerke R² of 0.83 indicates unusually high explanatory power for a clinical/forensic dataset. This may partly reflect the very large between-group difference in psychopathy (PCL-R), which can yield near-complete separation and inflate apparent model fit in logistic regression, particularly in modest samples. Accordingly, potential risks of overfitting, multicollinearity, and (quasi-)separation should be considered. Future work should evaluate model stability through internal validation (e.g., cross-validation/bootstrapping), report discrimination and calibration indices (AUC, calibration slope/Brier score), and consider penalized approaches (e.g., Firth logistic regression) to mitigate small-sample bias and separation effects.

### Strenght and limitations

This study has several notable strengths. It represents one of the few investigations to directly compare violent and sexual offenders formally diagnosed with ASPD using a comprehensive forensic-psychiatric dataset spanning ten years. The inclusion of multiple validated psychometric instruments—such as the PCL-R, MMPI, Beck inventories, and cognitive distortion scales—enhanced the multidimensional assessment of personality, affective, and cognitive domains relevant to offending behavior. Additionally, the integration of clinical, sociodemographic, and judicial data provided a robust framework for identifying distinct psychological and behavioral profiles within a high-risk forensic population. Despite this strengths, this study has several limitations. The cross-sectional design precludes causal inference, and the sample is limited to institutionalized offenders, restricting generalizability. Self-report measures such as the MMPI and Beck inventories may be subject to response biases. Additionally, some potentially relevant variables, such as neurobiological markers or detailed developmental histories, were not available. Future research should employ longitudinal and multimodal designs integrating psychometric, neurocognitive, and neuroimaging data to further elucidate pathways from personality pathology to specific offending patterns. Additionally, the Interpersonal Cognitive Distortions Scale (ICDS) was originally developed and validated in a non-forensic sample of university students. Therefore, its psychometric properties and interpretability in forensic populations may be limited, and findings related to cognitive distortions should be interpreted with caution.

## Conclusion

In conclusion, this study identified distinct clinical and psychological profiles between violent and sexual offenders with ASPD. Violent offenders demonstrated higher levels of psychopathy, cognitive distortions, substance use, and externalizing behaviors, whereas sexual offenders exhibited greater internalizing symptoms and more frequent histories of sexual trauma. Sociodemographic variables did not significantly differentiate the two groups, underscoring the dominant role of clinical and psychological factors in forensic risk assessment. These findings highlight the necessity of individualized, mechanism-focused interventions—such as aggression regulation and cognitive restructuring for high-psychopathy violent offenders, and affect regulation and trauma-focused therapies for sexual offenders—while emphasizing that incorporating psychopathy and cognitive schema assessments into forensic evaluations may improve both predictive accuracy and rehabilitation outcomes.
